# Percutaneous Stent-Graft Repair of Anastomotic Pseudoaneurysms following Vascular Bypass Procedures: A Report of Two Cases

**DOI:** 10.1155/2013/124832

**Published:** 2013-01-10

**Authors:** John Rundback, James Haug, Kevin Herman, Joseph Manno, Martin Cerda

**Affiliations:** Holy Name Medical Center, Teaneck, NJ 07776, USA

## Abstract

Anastomotic pseudoaneurysms are common entities following vascular bypass procedures and, if left untreated, serious complications such as thromboses, infection, and rupture can frequently occur. Therefore, attempts to employ various methods of repair have been utilized in treating anastomotic pseudoaneurysms to maximize operational success and future risk reduction. Herein, the authors report two cases of anastomotic pseudoaneurysms which were repaired percutaneously utilizing a combination of strategies such as careful preoperational image planning, multiple commercially available devices, and secondary embolization techniques.

## 1. Introduction

Anastomotic pseudoaneurysms (aPSAs) most commonly arise from late disruptions of arterial graft anastomoses and are rare complications associated with arterial bypass procedures. Causes of aPSA's are postoperative infection, suture fatigue, poor suture material, postoperative nicotine use, recurrent operations to the same site, trauma, and mechanical obstruction [1]. While aPSA's may occur in a variety of locations, the most frequent location is at a femoral artery graft anastomoses [2]. Current studies have theorized that this is due to turbulent blood flow that progressively weakens the arterial wall of end-to-side anastomoses promoting the development of anastomotic leaks [3]. Although aPSA's involving femoral bypass grafts are the most frequent location, the prevalence is low, occurring in 0.8–2.2% of revascularization procedures [4, 5]. 

The surgical management of aPSA's historically consists of resection of the initial graft with reimplantation of a new bypass either into the original arteriotomy or to a more distal target. An alternative is to resect the graft and place a Dacron tube interposition graft between the original graft limb and the host vessel [6]. While these procedures have a high success rates, secondary open surgical procedure still poses higher risk than a primary operation. Re-exploration can be difficult due to anatomical distortion and postoperative scarring, supporting a potential value for an endovascular strategy when possible. 

We have recently experienced two cases that involved solely endovascular repair of aPSA's, the first at the proximal anastomosis of an axillofemoral bypass, and the second a femoral aPSA that formed following a bilateral axillofemoral graft bypass procedure. Both cases were successfully treated percutaneously with stent grafts to exclude flow into the aPSA's.

**Case 1**

**Figure 1:**
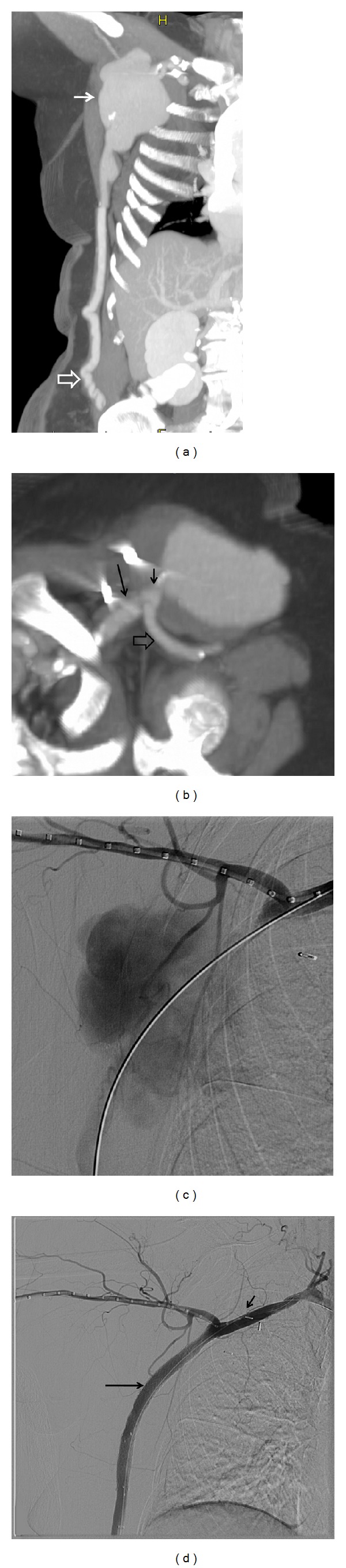
(a) Coronal CT scan demonstrates a large aPSA near the proximal end of an axillofemoral bypass graft (arrows). The distal end of the bypass is noted by the open arrow. (b) A reconstructed para-axial image from the CT scan better shows the relationship between the aPSA and proximal bypass anastomosis. Long arrow corresponds with the axillary artery with the open arrow pointed at the axillary vein. A short landing zone for stent graft exclusion is noted at the neck of the aPSA (short arrow). (c) Angiogram shows filling of a large aPSA located 2 cm from the subclavian artery anastomosis. A marker catheter has been introduced from the brachial access and a wire traverses the graft and extends into the proximal subclavian artery via a direct distal bypass puncture. (d) Successful complete exclusion of PSA is seen on completion arteriography with preserved flow through both the native subclavian (short arrow) and brachial artery as well as the axillobifemoral graft (long arrow).

An 80-year-old female patient with a past medical history significant for hypertension, hyperlipidemia, atherosclerotic heart disease, and peripheral vascular disease presented 12 years after an axillobifemoral bypass graft procedure with an enlarging and painful right axillary mass which was confirmed by chest CT to be a large aPSA measuring 8.2 × 5.0 × 9.8 cm (Figure 1(a)). Ultrasound of the mass also revealed a mural thrombus at the junction of the right host axillary artery and the graft, with the graft still patent. There was also a smaller aneurysm evident at the distal anastomosis measuring 2.7 cm in the right groin. The patient had no trauma or known intercurrent illness or infection contributing to the aPSA formation. Given the patient's age and co-morbidities, it was decided to attempt endovascular exclusion. Multiple orthogonal reconstructions of the CTA data set were performed to allow the demonstration of a suitable proximal landing zone for stent-graft placement (Figure 1(b)), and to develop a plan for placement of a diagnostic catheter from an arm approach and direct bypass graft puncture for definitive treatment. Ultrasound guided needle access was performed both in the brachial artery as well as the lower aspect of the bypass graft. A 4-French sheath was placed into the brachial artery, and a 6-French and subsequently 10-French sheath was inserted into the bypass graft. Heparin 5000 units was given intravenously. A marker catheter (Cook, Bloomington, IN) was advanced across the proximal origin of the bypass form the arm approach for diagnostic arteriography, allowing the advancement of a braided steerable catheter (Kumpe catheter, Angiodynamics, Bloomsbury, NY) and 0.035′′ angled glidewire (Terumo, Somerset, NJ) via the graft sheath across the aPSA and into the subclavian artery proximal to the bypass. Angiography demonstrated a large aPSA with a focal proximal neck arising approximately 2 cm from the subclavian anastamosis (Figure 1(c)). The brachial catheter was kept in position extending across the graft origin to mark the location of the artery and preserve access through the subclavian artery. From the graft approach, nested polytetrafluoroethylene (PTFE) covered stent grafts were deployed, with a 10 mm diameter I-Cast stent (Atrium, Hudson, NH) placed at the cranial aspect, an intervening 10 mm diameter by 10 cm long VIATORR graft (Cook) placed into the iCAST, and then a second iCAST stent placed at the bottom. Angiography demonstrated a small amount of residual PSA flow for which a 10 mm Fluency stent (Bard, Tempe, AZ) was placed below and extending the coverage of the second I-Cast. All stents were post dilated with a 10 mm balloon. Completion arteriography both before and after removal of the wires showed successful complete exclusion of PSA filling with preserved flow through both the native subclavian and brachial arteries as well as the axillo-bifemoral graft (Figure 1(d)).

The bypass sheath was removed and hemostasis was achieved with a Perclose device (Abbott Laboratories, Abbott Park, Illinois). Since the patient was anti-coagulated, an attempt was also made to use a Perclose device for closure at the brachial sheath site. However, this resulted in a loss of distal radial and ulnar pulses for which surgical repair of the brachial artery was performed. A subsequent CT scan showed successful exclusion of the aPSA, and the patient has remained asymptomatic during 8 months of follow-up.

**Case 2**

**Figure 2:**
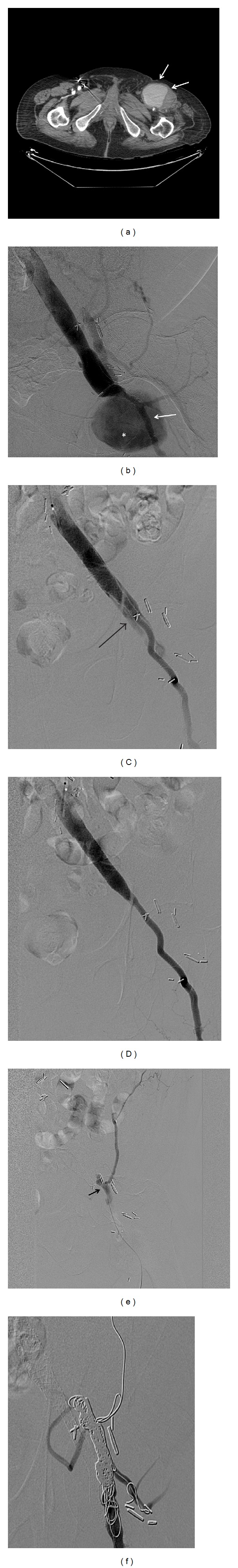
(a) A CT scan shows a large vascular mass in the left groin (arrows) consistent with an aPSA in this patient with an aortobifemoral bypass. (b) Angiography of the left iliac limb of the ABF via a brachial approach shows a left femoral aPSA (asterisk). Note the small caliber atherosclerotic profunda femoral artery (arrow) as well as SFA occlusion. (c) Follow-up angiogram after initial self-expandable and sequentially dilated balloon expandable stent-grafts shows diminished filling of the aPSA with persistent perigraft leak (arrow). (d) Repeat angiography following additional larger dilation of the cephalad iCast stent graft now shows nonfilling of the aPSA. (e) An angiogram performed via direct puncture of the sPSA after iliac and profunda femoral stent-graft placement shows communication between the pseudoaneurysmal sac and the EIA (arrow). (f) Angiography after coil embolization of the EIA. A subsequent ultrasound showed complete exclusion of flow in the aPSA.

A 74 year-old male patient with PAD, a prior aorto-bifemoral bypass (ABF) 5 years earlier, and an above knee left leg amputation presented to the emergency room with right leg pain and absent pulses. Cardiovascular risk factors included hypertension, hypercholesterolemia, coronary artery disease, prior MI and congestive heart failure. A CTA showed occlusive femoral disease on the right with reconstitution at the popliteal level and revealed an incidental 4.5 cm enhancing mass located in the left groin consistent with an aPSA. At the discretion of the patients vascular surgeon, no intervention was performed at this time, and the patient was lost to follow-up. Eight months later, the patient represented with discomfort in the left groin and an enlarged pulsatile mass. CTA was repeated demonstrating enlargement of the PSA which now measured approximately 8 cm (Figure 2(a)), occlusion of the native SFA, and a small caliber profunda femoral artery. The patient was referred for angiography and endovascular intervention.

Via a left brachial puncture, angiography of the left iliac limb of the ABF was performed showing filling of the pseudoaneurysm sac, reflux into the terminal portion of the native left external iliac artery (EIA), and confirmed occlusion of the native superficial femoral artery (SFA) with a a small diseased profunda femoral artery (Figure 2(b)). Via a 7 French brachial sheath, two 6 mm × 100 mm overlapping Viabahn stent grafts (Gore, Flagstaff, AZ) were initially deployed extending from the profunda femoris artery into the left limb of the ABF. Follow-up angiograms showed continued perigraft filling of the PSA. Therefore, a total of three nested 8 mm nominal diameter iCast stent grafts (Atrium medical, Hudson, New Hampshire) were sequentially placed and post dilated to 10 mm. The ICast grafts were position to overlap each other and the Viabahn graft by approximately 1 cm. Follow-up angiograms showed decreased but continued aPSA filling due to incomplete apposition of the graft to the ABF limb. (Figure 2(c)).

At this point, the plan was to further dilate the 10 mm iCast stent grafts to 14 mm. However, we did not initially have 14 mm balloon on a sufficiently long shaft to reach the femoral area from the brachial puncture. Upsizing to a 10 French brachial sheath to accommodate a larger diameter Viabahn was considered but not performed due to a concern for brachial artery occlusion. Unsuccessful attempts were made advance a sheath across the aorto-bifemoral bifurcation from a right femoral puncture, and it was decided to terminate the procedure and stage a second transbrachial intervention after obtaining larger diameter longer shaft balloon. Puncture of the profunda femoral artery was also considered to allow addition balloon dilation from a retrograde approach, but was decided against because it was felt that this might compromise or thrombose the solitary profunda runoff. Hemostasis was successfully achieved after sheath removal with manual pressure without complication.

The patient was brought back to the angiography suite the following day, and a high brachial puncture (approximately four finger-breadths from the axillary crease) was again performed, allowing placement of a 7 French sheath. Further PTA to expand and dilate the most cephalad iCast stent graft was performed with 14 mm balloon catheter, with angiography showing nonfilling of the aPSA (Figure 2(d)). However, since initial imaging showed communication of the PSA with the remnant patent distal native EIA, it was felt that EIA occlusion was necessary for complete exclusion of the aPSA. Therefore, the sac was punctured with ultrasound guidance a 4 French sheath was inserted and the native external iliac artery was catheterized with a short angled 4 French catheter (Kumpe, Angiodynamics, Bloomsbury, NY). Angiogram showed flow into the pseudoaneurysm sac from the EIA (Figure 2(e)), and coil embolization of the EIA was performed with multiple 0.035′′ fibered stainless steel Azure coils (Terumo, Somerset, NJ) (Figure 2(f)). Occlusion of the aneurysm sac was further performed with the injection of 2 cc of 1 : 1000 topical thrombin (Ethicon, Somerville, NJ). A four month follow up CT scan without contrast (due to renal insufficiency) showed significant decrease in the pseudoaneurysm sac size and there was no flow on duplex ultrasound.

## 2. Discussion

The cumulative risk of clinically significant aPSA's is small, ranging from 2–6% of cases [7]. Enlarging or symptomatic aPSA's warrant intervention due to an untreated risk of thrombosis, infection, or rupture [8]. Given the inherent difficulties of surgical control due to prior dissection and adhesions at the anastomotic site [9], as well as the potential for graft loss due to flow occlusion during surgical revision, minimally invasive strategies utilizing direct puncture or endovascular approaches are appealing if anatomically possible. Nonsurgical techniques minimize operative blood loss, lower cardiac and pulmonary complications, reduce anesthetic and transfusion requirements, and allow for a rapid postprocedural recovery. 

Nonoperative strategies for the treatment of femoral PSA's occurring after arterial puncture alone have been described, and consist of ultrasound guided compression with or without thrombin injection [10], and transcatheter management with coils and/or stent-grafts. However, ultrasound guided compression has not to our knowledge been described for aPSA's, and in general would not be feasible in large PSA's or those having a patulous connection with the underlying arterial structures. Similarly, thrombin injection alone is potentially risky due to the anticipated wide communication between the PSA and bypass or artery in aPSA's, with an associated substantial risk of native arterial thrombosis due to incomplete containment of thrombin within the PSA. Coil embolization is well established for the treatment of PSA's in multiple vascular territories, but is dependent on the presence of appropriate anatomy. In fusiform type PSA's or those with broad necks, there may be considerable difficulty obtaining satisfactory closure of the PSA while preserving flow in the parent artery. For PSA's with this morphology, the endovascular placement of commercially available stent grafts potentially allows reliable exclusion of the PSA while maintaining arterial perfusion to distal vascular beds. Prior studies have advocated stent-graft exclusion of aPSA's following arterial bypass procedures with promising long term results [8, 11]. These reports have described preserved patency at 2 to 3 year following stent-graft placement for sPSA's involving both aortofemoral and aortoiliac bypass grafts [8, 11]. 

We feel that several notable points are elucidated in these cases. First off, vascular imaging prior to intervention is extremely important in determining the suitability for endovascular repair compared with other strategies, and is enhanced by the ability to perform multiplanar reconstructions of axial data sets to accurately measure the parent vessel diameter as well as proximal and distal landing zone lengths in cases in which stent graft management is being considered. Image planning also is critical for assessing the access site and approach for treatment. This is well demonstrated in both of our cases. Oblique reformations of the CT images in the patient with the axillofemoral graft PSA were necessary to reveal a short but sufficient length proximal landing zone for stent-graft exclusion, and guided the planning for placement of a small caliber sheath and marker imaging catheter from the brachial approach and larger sheath for stent-graft insertion via a graft puncture. The finding of SFA occlusion and a small profunda femoral artery in the patient with the ABF distal aPSA mandated an initial transbrachial approach as well.

Secondly, the complexity of aPSA's potentially requires the use of multiple devices and approaches for a successful outcome. In both cases, multiple types of stent grafts with different performance characteristics were used in combination. Self expanding stent grafts such as the Viabahn or Fluency allow optimal conformance to underlying vessels, whereas a balloon-expandable stent graft such as the iCast affords higher radial strength, the ability for post-dilation and upsizing after implantation, and possibly a better seal if there is a short proximal or distal landing zone. The availability of a wide range of devices in the interventional suite as well as the need for comfort and familiarity with each device cannot be overstated.

Finally, PSA's occurring at the anastomoses of anatomic arterial bypasses such as an ABF graft intrinsically have dual supply from both the surgical graft inflow/outflow tract and the native proximal artery. Recognition of this is critical to allow targeted treatment of both routes of continued PSA filling. While stent grafts can reliably and durably occlude flow into the PSA via the major conduit, secondary embolization of the (bypassed) native arterial inflow is an important adjunctive treatment.

## 3. Conclusion

Anastomotic PSA's following surgical arterial bypasses are rare but often amenable to endovascular therapy. Stent-grafts provide a safe, effective, and durable option in these cases while allowing preservation of flow in the surgical graft. Careful planning using cross-sectional imaging, the availability of several different commercially available devices, and secondary techniques to assure complete exclusion of the PSA may be necessary for successful management.

## Abbreviation

EIA: external iliac artery
aPSA: anastomotic pseudoaneurysm
SFA: superficial femoral artery
ABF: axillobifemoral bypass.
